# The Average Body Surface Area of Adult Cancer Patients in the UK: A Multicentre Retrospective Study

**DOI:** 10.1371/journal.pone.0008933

**Published:** 2010-01-28

**Authors:** Joseph J. Sacco, Joanne Botten, Fergus Macbeth, Adrian Bagust, Peter Clark

**Affiliations:** 1 Department of Medical Oncology, Clatterbridge Centre for Oncology, Merseyside, United Kingdom; 2 Department of Clinical Oncology, Velindre Cancer Centre, Cardiff, United Kingdom; 3 Liverpool Reviews and Implementation Group, University of Liverpool, Liverpool, United Kingdom; CIET, Canada

## Abstract

The majority of chemotherapy drugs are dosed based on body surface area (BSA). No standard BSA values for patients being treated in the United Kingdom are available on which to base dose and cost calculations. We therefore retrospectively assessed the BSA of patients receiving chemotherapy treatment at three oncology centres in the UK between 1^st^ January 2005 and 31^st^ December 2005.

A total of 3613 patients receiving chemotherapy for head and neck, ovarian, lung, upper GI/pancreas, breast or colorectal cancers were included. The overall mean BSA was 1.79 m^2^ (95% CI 1.78–1.80) with a mean BSA for men of 1.91 m^2^ (1.90–1.92) and 1.71 m^2^ (1.70–1.72) for women. Results were consistent across the three centres. No significant differences were noted between treatment in the adjuvant or palliative setting in patients with breast or colorectal cancer. However, statistically significant, albeit small, differences were detected between some tumour groups.

In view of the consistency of results between three geographically distinct UK cancer centres, we believe the results of this study may be generalised and used in future costings and budgeting for new chemotherapy agents in the UK.

## Introduction

Body surface area (BSA), despite well-documented limitations, remains the most frequently used measure for calculating the dose of cytotoxic drugs in chemotherapy regimens[1], [2]. The dosing of cytotoxic drugs has been based on the observation that physiologic variables related to drug metabolism and elimination, such as basal metabolic rate, renal function and hepatic function, vary between individuals according to surface area[3]. This type of dosing calculation has traditionally been thought to reduce the variability in drug exposure between patients. However more recent research has questioned the reliability of BSA-based prediction of drug clearance, since the same dose of drug (per m^2^) frequently results in very different pharmacokinetic profiles and toxicity in different patients[4], [5]. An example is the substantial interpatient variability of etoposide clearance (coefficient of variation of 30%) observed in a study by Ratain et al[6].

The most widely used formula for BSA calculation is that devised by Du Bois and Du Bois in 1916. Plaster of Paris moulds of nine subjects were cut into small pieces in an attempt to measure the two-dimensional surface area of the skin. Each individual's body/skin surface area was then calculated and Du Bois and Du Bois determined that BSA was related to height and weight by the formula: weight (kg) ^0.425^×height (cm) ^0.725^×0.007184[7]. Notably, this formula was derived from 9 subjects only, one of whom was a child. The age, sex and nutrition of these subjects, studied in the middle of the First World War, are unlikely to be comparable to that of patients receiving chemotherapy in 2009. However, although several other formulae have since been put forward[8], [9], [10], none of these has gained widespread acceptance.

Drug dosage is usually determined by multiplying the patient's BSA by a constant that has been derived for each drug in phase 1 and 2 studies. Despite calls for BSA dosing to be replaced with dosing based on pharmacokinetics or pharmacodynamics, these new methods are yet to be agreed upon[5], [11]. The few exceptions include carboplatin dosing which is based on creatinine clearance[12] and folinic acid rescue following methotrexate infusion which is based on serum methotrexate levels.

With the development of new, usually costly chemotherapy and immunotherapy drugs, the commissioners and providers of cancer care as well as the National Health Service (NHS) as a whole, need to estimate how much a particular therapy will on average cost per year. Such estimates rely in part on an accurate assessment of body surface area. Although a mean BSA of 1.73 m^2^ has been quoted in previous work [13], this historical value is unlikely to represent patients currently being treated in the United Kingdom (UK), and does not take into account gender specific differences or recent increases in obesity in the general population.

More recent studies performed outside the UK provide some guidance. An Australian study[2] of 2838 patients receiving chemotherapy between May 1996 and December 2000 arrived at an overall mean BSA of 1.80 m^2^ (female 1.70 m^2^, male 1.89 m^2^), while in an international retrospective audit of 1650 patients on phase I trials between 1991 and 2001, Baker et al [14] reported a BSA of 1.86 m^2^. Notably, the latter study potentially represents a fitter group of patients with a higher BSA than the general population.

The lack of a standard BSA value on which to base these dose and cost calculations is reflected in the varying values used by the National Institute for Health and Clinical Excellence (NICE) in the appraisal of new agents. For instance, in recent guidance on the administration of cetuximab and bevacizumab to patients with metastatic colorectal cancer a mean BSA of 1.75 m^2^ was used[15]. However, the evidence review group for the use of cetuximab for patients with squamous cell carcinomas of the head and neck[16] used an average BSA of 1.7 m^2^. In an appraisal of pemetrexed for the second line treatment of non-small cell lung cancer, the manufacturers assumed a mean BSA of 1.7 m^2^ while the evidence review group used a BSA of 1.83 m^2^ [17]. Although these values appear quite similar, small differences can be of great significance as they may have an effect on the part use of an additional vial of chemotherapy leading to potential waste and therefore increased cost.

Dose banding, whereby prescribed chemotherapy doses are rounded up or down to pre-determined standard doses, is a common practice including in the three centres studied. In some malignancies, this reduces waste, treatment delays and minimises errors in making up unusual volumes of chemotherapy agent[2], [18]. Dose banding is almost invariably performed within a 5% tolerance limit and therefore does not result in a significant change to the administered dose.

This study was designed to establish the mean BSA of patients receiving chemotherapy in the UK and whether there are statistically significant differences in the BSA values for:

- Typeface="12";patients in different parts of the country
- Typeface="12";men and women
- Typeface="12";patients with different tumour types
- Typeface="12";patients receiving adjuvant chemotherapy and those receiving palliative or second line treatment.

With this information, more accurate estimates can be made of the average dose of an agent and the likely consequent cost to a service of expensive drugs.

## Methods

### Ethics Statement

The study did not require patient consent or approval by an ethics committee, as no intervention was involved, data were anonymised and no patient identifying information was included.

A retrospective study was performed assessing patients receiving chemotherapy treatment in three cancer centres in different areas of England and Wales–the Clatterbridge Centre for Oncology in Merseyside, Velindre Cancer Centre in Cardiff and the Dorset Cancer Centre in Poole.

In each of these centres, data were collected on patients who started a chemotherapy regimen between 1^st^ January 2005 and 31^st^ December 2005. Patients receiving chemotherapy for the following cancers were included: head and neck, ovarian, lung, upper GI/pancreas, breast and colorectal. All intravenous and oral chemotherapy regimens were included except single agent carboplatin because the dose calculation for this drug is independent of BSA. Immunotherapies, intrathecal and intrapleural chemotherapies were also excluded. No minimum age was applied.

In Poole and Cardiff, where records of all chemotherapy prescriptions are kept electronically, it was possible to obtain all the relevant data by exporting patients' details from computerised chemotherapy programmes (Clinichemo and ChemoCare). A proportion of the lung cancer patients in Cardiff received their chemotherapy at Llandough Hospital and data about height, weight and BSA were obtained from the local tumour-specific database.

In Clatterbridge, chemotherapy prescriptions are not recorded electronically and so only demographic data could be obtained electronically from the MAXIMS database. Height, weight and BSA were obtained from the original chemotherapy scripts, which are filed with the patients' clinical records.

The age, sex, height and weight for each patient were recorded. The BSA on their chemotherapy prescription was also noted. If a patient started more than one chemotherapy regimen during the 12 month period, the data were collected for each regimen because their weight and hence BSA might have changed. Treatment intent (i.e. neo/adjuvant or palliative) was also recorded for all patients with breast or colorectal cancer. Only a small percentage of patients received neoadjuvant chemotherapy, and these cases were included in the adjuvant group for analysis.

The above data were entered into Excel spreadsheets. We recalculated the BSA for each patient using the Dubois and Dubois method (BSA (m^2^) = Wt (kg) ^0.425^×Ht (cm)^0.725^×0.007184) and this value was used for further calculations. This was done to verify the accuracy of the original calculation and to remove any dose capping.

Statistical analyses were performed using SPSS version 15. All significance testing employed an independent sample Student's t-test, and was 2- tailed.

## Results

**Figure 1:**
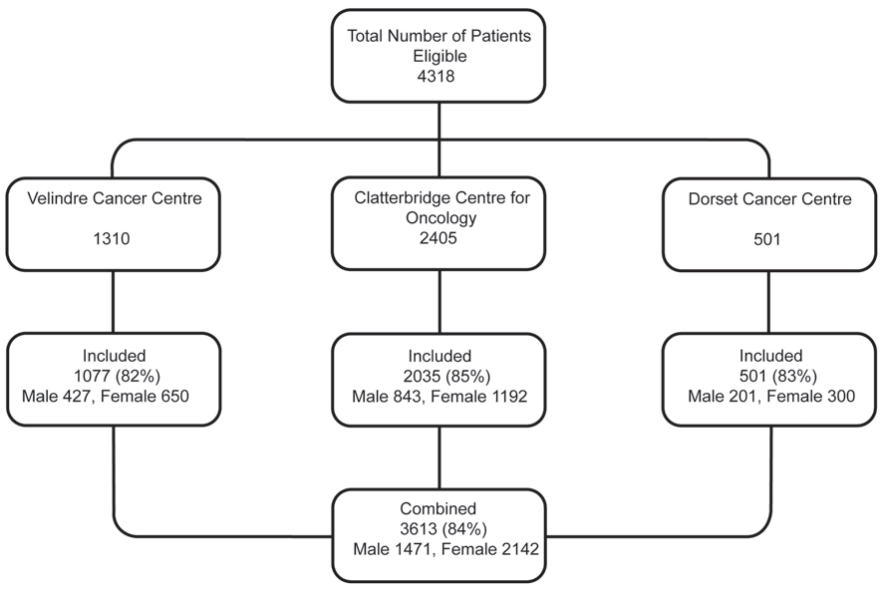
Patient identification and inclusion in study. Only cases in which a full dataset could be obtained were included in the study. This was possible for over 80% of patients in all 3 centres, with only a relatively small number excluded, mainly due to misfiling or loss of chemotherapy scripts.

A total of 4318 patients were identified as meeting the eligibility criteria. A full data set was obtained for 3613 patients (84%), and over 80% of patients from each centre were included (figure 1). The remaining patients were excluded because a complete data set was not available due to inaccurate filing and missing or unavailable records. Table 1 illustrates the demographic data for patients included in the study.

**Table 1:** 

	Velindre	Clatterbridge	Dorset	Combined
Number eligible	1310	2405	603	4318
Included (%)	1077 (82)	2035 (85)	501 (83)	3613 (84)
Median Age	61	60	65	61
Male (%)	427 (39.6)	843 (41.4)	201 (40.1)	1471 (40.7)
Female (%)	650 (60.4)	1192 (58.6)	300 (59.9)	2142 (59.3)

**Figure 2:**
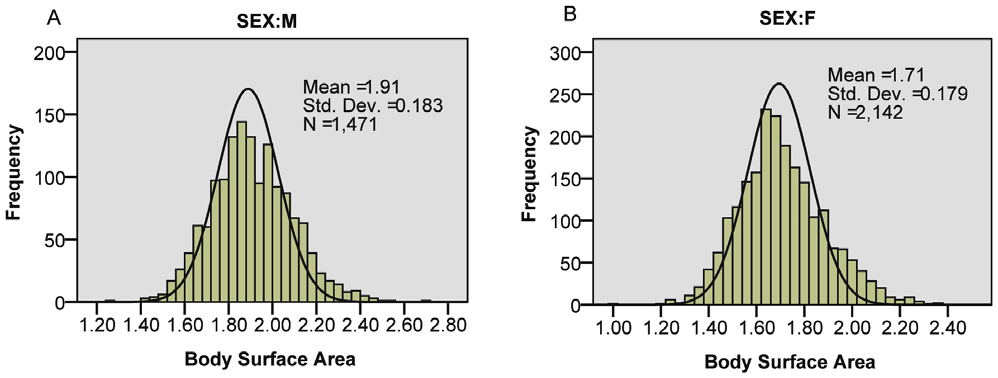
Histograms depicting the distribution of body surface area in men and women undergoing chemotherapy. As illustrated, the distribution approximates normal in both sexes.

The overall mean BSA for the patient population was 1.79 m^2^ (95% CI 1.78, 1.80). For both sexes, the BSA distribution was approximately normal as shown in figure 2. The mean BSA for men was 1.91 m^2^ (1.90, 1.91) compared to1.71 m^2^ (1.70, 1.72) for women, with a median of 1.90 and 1.70 respectively. Previous studies have employed the mean in preference to the median, a convention we have continued as the distribution approximated normal, and the mean better incorporates outliers which may have significant effects on costing.

**Figure 3:**
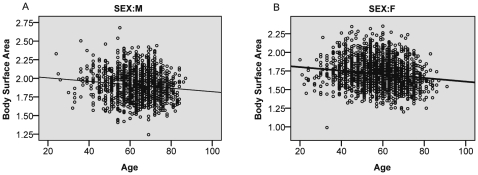
Correlation between age and BSA. The BSA was negatively correlated with age in both genders as shown in the scatterplots above. The Pearson correlation coefficient was −0.124 and −0.157 respectively and both results were statistically significant (p<0.0005).

The difference in mean BSA between male and female patients was statistically significant (p<0.0005), and apparent in all sub-groups, as shown in Table 2. In both sexes, there was a weak negative correlation between BSA and age as shown in figure 3. A breakdown of BSA data for each tumour site at each oncology centre is shown in Table 3. In each tumour site, the mean BSA is consistent across the three centres with overlapping confidence intervals.

**Table 2:** 

		Male	Female
Adjuvant Breast	n	4	685
	Mean (CI)	2.10 (1.59,2.61)	1.75(1.74,1.77)
Palliative Breast	n	4	282
	Mean (CI)	2.06 (1.85,2.28)	1.74 (1.72,1.76)
Adjuvant Colon	n	335	210
	Mean (CI)	1.92 (1.90,1.94)	1.68 (1.65,1.70)
Palliative Colon	n	291	151
	Mean (CI)	1.93 (1.91,1.96)	1.68 (1.65,1.71)
Head and Neck	n	117	38
	Mean (CI)	1.85 (1.81,1.88)	1.65 (1.59,1.72)
Lung	n	390	331
	Mean (CI)	1.89 (1.87,1.91)	1.65 (1.64,1.67)
Ovarian	n	0	321
	Mean (CI)	-	1.71 (1.69,1.73)
Upper GI	n	330	124
	Mean (CI)	1.90 (1.88,1.92)	1.65 (1.62,1.69)
Combined	n	1471	2142
	Mean (CI)	1.91 (1.90, 1.91)	1.71 (1.70, 1.72)

**Table 3:** 

		Velindre	Clatterbridge	Dorset	Combined
Adjuvant Breast	n	207	430	52	689
	Mean (CI)	1.76 (1.73,1.78)	1.76 (1.74,1.78)	1.74 (1.69,1.78)	1.76 (1.74,1.77)
Palliative Breast	n	80	170	36	286
	Mean (CI)	1.74 (1.69,1.78)	1.75 (1.72,1.77)	1.73 (1.67,1.79)	1.74 (1.72,1.76
Adjuvant Colon	n	158	308	79	545
	Mean (CI)	1.81 (1.78,1.84)	1.84 (1.81,1.86)	1.81 (1.76,1.86)	1.83 (1.81,1.84)
Palliative Colon	n	186	214	42	442
	Mean (CI)	1.83 (1.80,1.86)	1.86 (1.82,1.89)	1.86 (1.80,1.92)	1.85 (1.83,1.87)
H & N	n	43	67	45	155
	Mean (CI)	1.75 (1.68,1.82)	1.83 (1.78,1.88)	1.80 (1.75,1.86)	1.80 (1.77,1.83)
Lung	n	154	486	81	721
	Mean (CI)	1.77 (1.73,1.80)	1.78 (1.76,1.80)	1.84 (1.80,1.88)	1.78 (1.77,1.80)
Ovarian	n	107	114	100	321
	Mean (CI)	1.72 (1.69,1.75)	1.71 (1.68,1.75)	1.71 (1.67,1.75)	1.71 (1.69,1.73)
Upper GI	n	142	246	66	454
	Mean (CI)	1.81 (1.78,1.84)	1.84 (1.82,1.87)	1.84 (1.78,1.88)	1.83 (1.81,1.85)
Combined	n	1077	2035	501	3613
	Mean (CI)	1.78 (1.77–1.79)	1.79 (1.79,1.80)	1.79 (1.77,1.81)	1.79 (1.78,1.80)

A comparison was made between the BSA for patients receiving neoadjuvant or adjuvant chemotherapy and those receiving treatment in the palliative setting for breast and colorectal carcinoma. In both instances there were no statistically significant differences (p = 0.323 and p = 0.152 respectively).

On the other hand, differences between tumour sites are apparent. The mean BSA for women with breast cancer was significantly higher than those for women with colorectal, head and neck, lung, upper GI and ovarian cancer (p<0.0005, 0.001, <0.0005, <0.0005 and 0.002 respectively). However, no statistically significant differences were observed among the latter five groups.

In men, a higher mean BSA was observed in those with colorectal cancer compared to those with upper GI, lung or head and neck cancer (p = 0.019, 0.002 and <0.0005 respectively). Men with head and neck cancer also had a significantly lower BSA than those with either lung or upper GI cancer (p = 0.026 and 0.010 respectively).

## Discussion

Recent media coverage continues to highlight the problems of introducing new, costly cancer chemotherapy agents into the NHS. Regulatory bodies in several countries, including the UK, routinely consider evidence of cost-effectiveness when deciding on reimbursement of new therapeutic agents. Evaluation of cost effectiveness as carried out by the NICE technology appraisal process[19] requires estimating the drug costs for the average patient, which for most chemotherapy drugs involves calculations based on expected BSA values.

The importance of using accurate data and appropriate calculation methods can be illustrated from experience in the UK, where NICE considered the merits of pemetrexed[17] and erlotinib[20] compared to docetaxel for the treatment of non-small cell lung cancer. In both cases the manufacturers assumed that doses of docetaxel could be calculated on the basis of a mean BSA of 1.7 m^2^ for all patients (male and female). The costs of pemetrexed were calculated on a similar basis (erlotinib in tablet form does not require dose adjustment according to BSA). On this basis, it appeared that the extra cost of switching to pemetrexed is £3,006 per patient and for erlotinib is £1,865 per patient.

However, the calculations employed a relatively low mean BSA, and did not take into consideration the effects of the distibution of BSA values in the population. The latter may have a significant effect on the final estimate of incremental cost, due mainly to effects on vial usage. Chemotherapy agents are frequently marketed in large vial sizes, leading to a stepwise rather than continuous increase in vials (and hence cost) with increasing BSA. Although vial sharing may help prevent wastage, this is only possible in relatively large centres for more common tumour types, and may be affected by the stability of the agent.

Using the BSA results for lung cancer patients in Table 2, and assuming 70% of such patients are male, the combined population mean BSA would be 1.818. A recalculation using this mean BSA and taking into consideration the distribution of BSA values is shown in Appendix S1. Incremental drug costs per patient would be £3,712 (23% more than originally proposed) for pemetrexed and £1,840 (1% less) for erlotinib. Differences of this order are likely to have important consequences for pharmacy budgets, and may be decisive in reimbursement decisions. Further discussion and examples of the utility of the data provided by this study are provided in Appendix S2, based on several NICE technology appraisals.

In the absence of reliable estimates of BSA distribution in UK adult patients with cancer, cost-effectiveness evaluations have previously depended on the use of data derived from studies conducted in other countries (table 4). In this study, we provide an estimate for the overall mean BSA for patients receiving chemotherapy at three centres in England and Wales. Additionally, we have analysed the BSA by geographical area, sex and tumour site. The results were consistent between the three geographical areas (North West England, South England and South Wales), and we therefore believe that these values can be extrapolated across the whole of the UK population.

**Table 4:** 

Area	Average Body Surface area
USA	1.86 m2 (Baker et al)
Not reported	1.73 m2 (Ratain)
Australia	1.80 m2 (Dooley et al)
UK	1.79 m2 (Sacco et al)

Unsurprisingly BSA varied with both sex and age. Men had a significantly higher BSA value than women (p<0.0005) and for both men and women the BSA declined with age (fig. 2). However the association between age and BSA was relatively weak (Pearson correlation coefficient of −0.124 and −0.157 for men and women respectively). Notably, the Dorset patients were older on average than those of the other two centres, but this was not reflected in significantly lower BSA values.

This study was designed to allow calculation of the mean BSA for patients with different tumour types. The tumour sites selected reflect those patients commonly treated with chemotherapy (such as breast cancer). On the other hand prostate cancer, which is primarily treated with hormone therapy was not included. This is reflected in the higher proportion of female patients in this study, as breast cancer and prostate cancer are the most common cancers in men and women respectively. We specifically concentrated on larger groups to eliminate the bias from small numbers of patients treated with chemotherapy for other tumour sites. We feel that most of the tumour site groups are large enough to allow generalisation of results although we acknowledge that we had data on comparatively few (155) head and neck cancer patients. Because of this criterion, we have included two tumour sites, breast and ovary, for which the patients are almost all female, which accounts for the higher proportion of female patients in this study.

It is commonly assumed that patients receiving palliative chemotherapy have a lower BSA than those receiving treatment in the neoadjuvant or adjuvant setting, because the more advanced tumours might be associated with significant anorexia and weight loss. We analysed data from two tumour sites (breast and colorectal carcinoma) to investigate this hypothesis. In both cases, there was no statistically significant difference between the mean BSA results, even though palliative chemotherapy included second and third line regimens. This might be due to stringent patient selection for palliative chemotherapy making it less likely that patients with significant weight loss received chemotherapy.

However, small but statistically significant differences were observed between some tumour groups. In particular, women with ovarian cancer had a significantly lower BSA than those with breast cancer and patients with lung cancer had a significantly lower BSA than those with colon cancer. In both cases this is presumably related to the well known association of both ovarian and lung cancer with significant weight loss and anorexia.

A maximum BSA of 2 m^2^ is commonly used for dose calculations in obese patients. This capping is based in part on small trials which indicate reduced clearance of some chemotherapy agents in obese patients[21], [22]. However the results of a recent pharmacokinetic study suggest that, for most evaluated drugs, full BSA should be used for dose calculation[23]. Additionally, recent studies in breast cancer have indicated dose capping may result in underdosing of some patients[24], [25]. For this reason we have not incorporated dose capping in our BSA calculations. However we have appended the raw BSA data for each tumour group (Appendix S3), thus allowing recalculation of BSA, taking capping into account. Notably dose capping at a BSA of 2 may have significant cost implications, as patients dosed on the basis of a BSA exceeding 2 would require a further vial. In our study 28% of male patients and 6.6% of female patients had a BSA over 2.00, and would potentially fall into this bracket. Assuming dose banding with 5% tolerance the proportion would be reduced to 14.5% for men and 2.4% for women.

This study provides a reliable estimate for the mean BSA of men and women receiving chemotherapy in the UK (1.91 and 1.71 respectively). While a tumour specific estimate may be more accurate for some tumour types, these differences were relatively small. This information will not only be of value to those calculating the future cost impact of new chemotherapy agents for which the dose is calculated from the BSA, but also to those estimating the cost-effectiveness of new and established agents.

## Supporting Information

Appendix S1 (0.05 MB XLS)

Appendix S2 (0.03 MB DOC)

Appendix S3 Body surface area raw data.
(0.58 MB XLS)
