# Supplementary material for: The Average Body Surface Area of Adult Cancer Patients in the UK: A Multicentre Retrospective Study
Source: PLoS One. 2010 Jan 28;5(1):e8933. doi: 10.1371/journal.pone.0008933 (PMC2812484; doi:10.1371/journal.pone.0008933)
Supplement: Appendix S2 — (0.03 MB DOC) [file pone.0008933.s002.doc]

*Effects of study results on reimbursement assessments*

The value of these results to healthcare policy may be seen from recent experience in reimbursement decisions in the UK. To date these survey data have been employed to improve drug cost estimates in four Single Technology Appraisals (STAs) of new chemotherapy indications carried out for the National Institute for Health and Clinical Excellence (NICE); three relating to non small cell lung cancer (NSCLC) [A1, A4, A5] and one to head and neck cancer [A3]. As described in the main text, average drug acquisition costs have been estimated very simply using a single average BSA value multiplied by the recommended dose per m^2^ and the list price per unit of drug (usually per mg). The data provided by this survey has allowed a more sophisticated approach to be used taking full account of cancer specific differences in mean BSA, gender differences in BSA, and also the effect of the wide distribution of BSA values in a patient population on individually prescribed doses. The separate and combined effects of each of these factors on the final estimated treatment cost are not always predictable, though in general the consequences for new agents tend to be greater than for established treatments, since the former normally enter the market at a premium price.

In early 2008, the application of this new approach for drug costing to the STA of pemetrexed as first-line chemotherapy for NSCLC [A1] led to apparently modest increases in the extra (incremental) drug cost of using pemetrexed compared to established agents of £165 - £215 per patient. However, this produced an important increase in the incremental cost-effectiveness ratio (ICER) of £6,800 - £8,900 per quality adjusted life-year (QALY) gained. (The NICE reference range [A2] considered cost-effective is £20,000 - £30,000 per QALY).

The STA relating to cetuximab in the treatment of squamous cell head and neck cancer [A3] saw increased cost estimates for both new and comparator regimens when the survey results were employed, but the higher list price of cetuximab led to an increase in the incremental cost per patient of £3,155 and an increase in the ICER of more than £22,000 per QALY.

**References**

A1 Fleeman N, Bagust A, McLeod C, Greenhalgh J, Boland A, Dundar Y, Dickson R. Tudur Smith C, Davis H, Green J and Pearson M. Pemetrexed for the first-line treatment of locally advanced or metastatic non-small cell lung cancer (NSCLC): A Single Technology Appraisal. LRiG, The University of Liverpool, 2009

<http://www.nice.org.uk/nicemedia/pdf/LungCancerAdvancedNSCPemetrexedEvidenceReviewReport.pdf> Accessed 3^rd^ December 2009

A2 Guide to the methods of technology appraisal. National Institute for Health and Clinical Excellence, June 2008

<http://www.nice.org.uk/media/B52/A7/TAMethodsGuideUpdatedJune2008.pdf> Accessed 3^rd^ December 2009

A3 Greenhalgh J, Bagust A, Boland A, Fleeman N, McLeod C, Dundar Y, Proudlove C and Shaw R. Cetuximab for metastatic and/or recurrent squamous cell carcinoma of the head and neck (SCCHN): A Single Technology Appraisal. LRiG, The University of Liverpool, 2008

<http://www.nice.org.uk/nicemedia/pdf/HeadNeckCancerCetuximabEvidenceReviewGroupReport.pdf> Accessed 3^rd^ December 2009

A4 Greenhalgh J, McLeod C, Bagust A, Boland A, Fleeman N, Dundar Y, Oyee J, Dickson R, Davis H, Green J, McKenna E, Pearson M. Pemetrexed for maintenance treatment of locally advanced or metastatic non-small cell lung cancer (NSCLC): A Single Technology Appraisal. Health Technology assessment (Winchester) 2010 in press.

A5 Brown T, Boland A, Bagust A, Oyee J, Hockenhull J, Dundar Y, Dickson R, Ramani VS, Proudlove C and McKenna E. Gefitinib for the first-line treatment of locally advanced or metastatic non-small cell lung cancer (NSCLC): A Single Technology Appraisal. Health Technology assessment (Winchester) 2010 in press.
